# Rare case of retroperitoneal mitotically active leiomyoma in a postmenopausal woman

**DOI:** 10.1093/jscr/rjad321

**Published:** 2023-06-05

**Authors:** Susumu Doita, Fumitaka Taniguchi, Kengo Mouri, Eiki Miyake, Minami Hatono, Hiroki Kajioka, Toshihiro Ogawa, Megumi Watanabe, Takashi Arata, Kou Katsuda, Kouji Tanakaya, Hideki Aoki

**Affiliations:** Department of Surgery, NHO Iwakuni Clinical Center, Yamaguchi, Japan; Department of Surgery, NHO Iwakuni Clinical Center, Yamaguchi, Japan; Department of Surgery, NHO Iwakuni Clinical Center, Yamaguchi, Japan; Department of Surgery, NHO Iwakuni Clinical Center, Yamaguchi, Japan; Department of Surgery, NHO Iwakuni Clinical Center, Yamaguchi, Japan; Department of Surgery, NHO Iwakuni Clinical Center, Yamaguchi, Japan; Department of Surgery, NHO Iwakuni Clinical Center, Yamaguchi, Japan; Department of Surgery, NHO Iwakuni Clinical Center, Yamaguchi, Japan; Department of Surgery, NHO Iwakuni Clinical Center, Yamaguchi, Japan; Department of Surgery, NHO Iwakuni Clinical Center, Yamaguchi, Japan; Department of Surgery, NHO Iwakuni Clinical Center, Yamaguchi, Japan; Department of Surgery, NHO Iwakuni Clinical Center, Yamaguchi, Japan

## Abstract

Leiomyomas are benign smooth muscle tumors, and retroperitoneal leiomyomas without coexisting uterine leiomyomas are extremely rare. Mitotically active leiomyomas, which are leiomyomas with increased mitotic activity, are rarely observed in postmenopausal women, except under the influence of exogenous hormones. This report presents a rare case of a retroperitoneal mitotically active leiomyoma in a postmenopausal woman. The patient presented with an abdominal mass and underwent surgical resection of the retroperitoneal tumor. Pathological examination revealed a mitotically active retroperitoneal leiomyoma with 31 mitotic figures per 10 high-power fields. The patient did not experience recurrence during the 2-year follow-up period. This case highlights the need to consider retroperitoneal mitotically active leiomyomas in postmenopausal women and suggests that myomectomy can prevent their recurrence.

## INTRODUCTION

Leiomyomas are benign smooth muscle tumors that represent the most common gynecological neoplasms [1]; however, primary retroperitoneal leiomyomas without coexisting uterine leiomyomas are rare [2]. If a small part of the leiomyoma exhibits increased mitotic activity, it is called a mitotically active leiomyoma. Mitotically active leiomyomas are rarely observed in postmenopausal women, except under the influence of exogenous hormones [3]. The clinical manifestations and management of mitotically active leiomyomas are similar to those of classical leiomyomas. In general, leiomyomas are curable by surgery; however, it is unknown whether retroperitoneal mitotically active leiomyomas are curable or not. Here, we report a rare case of a surgically removed retroperitoneal mitotically active leiomyoma in a postmenopausal woman, without recurrence 2 years after the surgical resection.

## CASE REPORT

**Figure 1:**
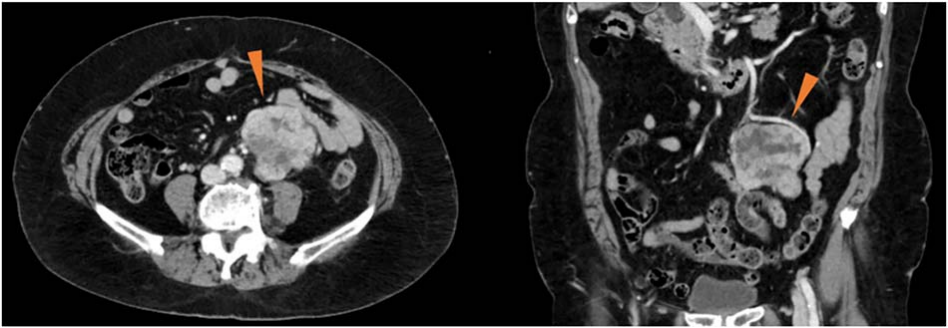
Contrast-enhanced computed tomography showing a well-defined, heterogeneously enhanced mass (red triangles) in the vicinity of the left ovarian artery and left ureter.

An 82-year-old woman with an abdominal mass was referred to our hospital. She had previously undergone ovarian mucinous cystadenoma with bilateral salpingo-oophorectomy 3 years prior, and had uterine leiomyoma. Abdominal contrast-enhanced computed tomography revealed a well-defined heterogeneously enhanced mass near the left ovarian artery and ureter (Fig. 1). No signs of metastases were observed. The serum tumor marker levels, including cancer antigen 19–9 and carcinoembryonic antigen, were within the reference ranges.

**Figure 2:**
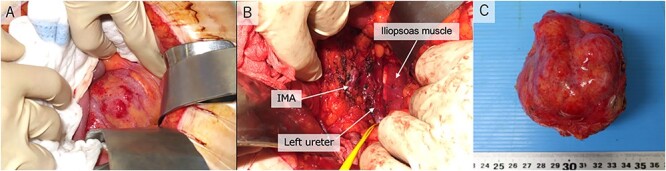
Operative pictures. (**A**) retroperitoneal tumor; (**B**) inferior mesenteric artery, iliopsoas muscle and left ureter after resection; (**C**) operative specimen IMA, inferior mesenteric artery.

During surgery, we observed a retroperitoneal tumor with a smooth surface at the back of the sigmoid mesentery (Fig. 2A). The tumor was firmly adherent to the mesentery. Therefore, we resected the retroperitoneal tumor and performed a partial colectomy (Fig. 2B). The left ovarian artery and vein were dissected as the vessels were connected to the tumor. The resected tumor was a well-circumscribed, firm, gray-white lobulated lesion with necrotic and mucinous changes (Fig. 2C). The patient’s postoperative course was uneventful, and she was discharged on the 19th postoperative day.

**Figure 3:**
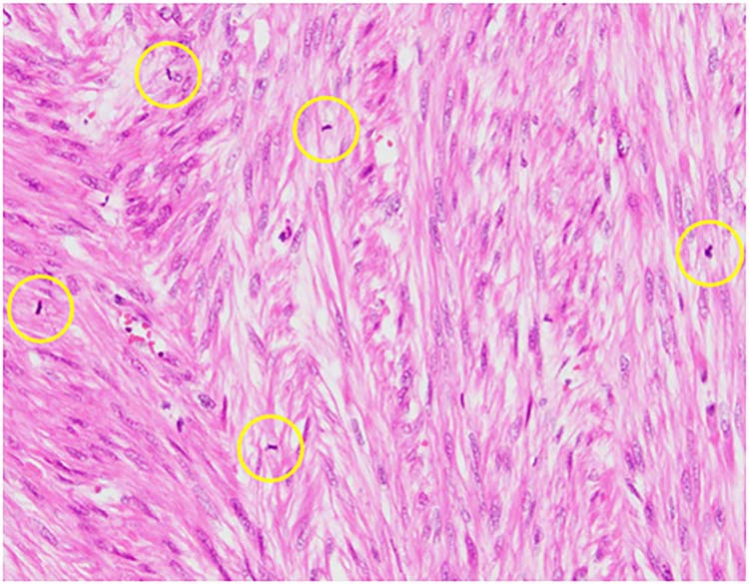
Hematoxylin–eosin staining, ×10 showing increased mitotic activity (yellow circles).

Pathological reports revealed a smooth muscle tumor with 31 mitotic figures per 10 high-power fields, but lacking cytologic atypia and tumor cell necrosis (Fig. 3). Based on the histological staining results, the patient was diagnosed with a retroperitoneal mitotically active leiomyoma.

The patient was doing well at the outpatient follow-up at 2 years, with no recurrence.

## DISCUSSION

The lessons learned from this case are as follows: (i) retroperitoneal mitotically active leiomyomas can occur in postmenopausal women and (ii) myomectomy does not result in tumor recurrence.

Retroperitoneal mitotically active leiomyomas can be observed in postmenopausal women. Leiomyomas are non-cancerous monoclonal tumors that arise from smooth muscle cells and fibroblasts that can exhibit various types of smooth muscle differentiation. Although various atypical localizations of leiomyomas have been reported, growth in the retroperitoneum is extremely rare [4]. Additionally, the etiopathogenesis of primary retroperitoneal leiomyomas has not been fully elucidated. In general, classic leiomyomas have the following features: a mitotic index of <5 mitotic figures per 10 high-power fields, mild cytologic atypia and no tumor cell necrosis. In our patient, the leiomyoma had 31 mitotic figures per 10 high-power fields, but lacked cytologic atypia and no tumor cell necrosis, which warranted the classification of mitotically active leiomyoma [5, 6]. Progesterone is thought to influence the mitotic index of smooth muscle tumors [7]; consequently, leiomyomas are more likely to have an increased mitotic count if they are excised during the secretory phase of the menstrual cycle or pregnancy, or when patients receive exogenous progestins [5, 8, 9]. The previous findings explain why mitotically active leiomyomas are rarely observed in postmenopausal women [3]. To the best of our knowledge, this is the first reported case of a retroperitoneal mitotically active leiomyoma in a postmenopausal woman.

Even after myomectomy of the retroperitoneal mitotically active leiomyoma, no recurrence or metastasis was observed during follow-up. In general, classic leiomyomas have the following features: mitotic index of <5 mitotic figures per 10 high-power fields, mild cytologic atypia and no tumor cell necrosis, whereas a diagnosis of leiomyosarcoma requires at least two of the following three features; mitotic index of ≥10 mitotic figures per 10 high-power fields, moderate to severe cytologic atypia or presence of tumor cell necrosis [10]. Mitotically active leiomyomas do not satisfy the histological criteria of being unequivocally benign or malignant. According to previous reports, the benign clinical behavior of such tumors warrants the classification of benign smooth muscle tumors rather than smooth muscle tumors of uncertain malignant potential or low-grade leiomyosarcoma [5, 11]. Several studies have reported smooth muscle tumors of the uterus, and the management of leiomyomas with increased mitotic activity was based on surgical treatment (myomectomy or hysterectomy with or without adnexectomy) [6, 7, 10, 12]. Hormonal stimulation, both endogenous and exogenous, may play a role in increasing the number of mitotic figures in mitotically active leiomyomas [13]. In the present case, increased mitotic figures were observed in the absence of progesterone during the postmenopausal period and in the retroperitoneum; therefore, the possibility of malignancy was considered to be higher than that of leiomyomas. Another case report described leiomyosarcoma arising in a patient with a prior mitotically active leiomyoma [14]. However, the retroperitoneal mitotically active leiomyoma in our patient did not recur for 2 years after myomectomy, which proved that surgical treatment (myomectomy) is useful for managing retroperitoneal mitotically active leiomyomas.

In conclusion, this case report indicated that mitotically active leiomyomas can occur after menopause and are curable by myomectomy without the need for postoperative chemotherapy or pelvic radiotherapy. Further studies are required to determine the optimal treatment for mitotically active retroperitoneal leiomyomas.

## Data Availability

Data sharing is not applicable to this article as no new data were created or analyzed in this study.
